# Quantitative immunoassay for mink immunoglobulin in serum and milk

**DOI:** 10.1186/s13028-018-0391-7

**Published:** 2018-06-18

**Authors:** Ronja Mathiesen, Mariann Chriél, Tina Struve, Peter Mikael Helweg Heegaard

**Affiliations:** 1 Innate Immunology Group, National Veterinary Institute, Technical University of Denmark, Kemitorvet Building 204, 2800 Kgs. Lyngby, Denmark; 2 Present Address: Innate Immunology Group, Department of Biotechnology and Biomedicine, Technical University of Denmark, Kemitorvet Building 204, 2800 Kgs. Lyngby, Denmark; 3 Division of Diagnostics & Scientific Advice-Diagnostic & Development, National Veterinary Institute, Technical University of Denmark, Kemitorvet Building 204, 2800 Kgs. Lyngby, Denmark; 4 Kopenhagen Fur, Langagervej 60, 2600 Glostrup, Denmark

**Keywords:** Mink (*Neovison vison)*, Milk IgG, Sandwich ELISA, Serum IgG, Validation

## Abstract

**Background:**

The significance of maternal immunoglobulin G (IgG) for the resistance against a number of infections affecting the health of young mink offspring is not known. Here, we present a validated immunoassay for quantification of mink IgG in serum and milk, using a commercially available polyclonal goat anti-ferret IgG antibody cross-reactive with mink IgG as both the catching and the detection antibody, in a sandwich format enzyme linked immunosorbent assay (ELISA). Using this ELISA, serum IgG concentrations was analyzed over time in both mothers and kits in order to establish a correlation between maternal IgG serum concentrations and those of the offspring.

**Results:**

Intra-assay coefficient of variation (CV) for a serum sample ranged from 2.15 to 5.97% depending on the dilution, while the inter-assay CV ranged from 5.17 to 17.78%. In addition, the range of milk intra-assay CV was 2.71–5.92%, while the range of the inter-assay CV was 4.20–16.03%. Calibrating the ELISA with purified mink IgG (an in-house preparation purified from mink serum) the lower limit of detection was found to be 5 ng/mL for serum and 1 ng/mL for milk. Both serum and milk showed high precision and good linearity over a two-log dilution range. When comparing the serum IgG concentrations of the mink kits a clear within litter effect was seen, while the mean serum IgG concentrations of litters differed significantly between some of the litters (P = 0.0013). Mean maternal serum IgG concentrations correlated positively with the IgG serum concentration of the corresponding offspring sampled over a 3 week period (R^2^ = 0.63).

**Conclusions:**

A calibrated and reproducible sandwich ELISA for quantifying mink IgG concentrations in both milk and serum with high analytical sensitivity was developed and validated. The results in this study corroborate previous investigations supporting the usability of the ELISA, paving the way for investigations into the importance of maternal IgG in milk and in serum for the welfare and health of the offspring.

## Background

Loss of mink kits during the pre-weaning period is of major concern for welfare, management, and economy in the mink fur production. Mink kits are very vulnerable to pathogens at this stage as they are born with very low serum concentrations of maternal immunoglobulin G (IgG), received through trans-placental transfer from the mother, and must strengthen their immune system by taking up IgG from the mother’s colostrum and milk [1, 2]. Furthermore, mink kits do not initiate production of IgG until they are 7–8 weeks old, leaving them vulnerable for the first few weeks of life [1, 3]. IgG absorbed from colostrum and milk play a critical role in passive immunization of mink kits against pathogens (for an extensive review on IgG transfer from mother to offspring see [4]). In ferrets, maternal immunoglobulins transferred through the milk were found to protect offspring against influenza virus infection [5, 6] and maternal IgG is likely to play a similar important protective role against infection in mink. The purpose of this study was to develop and validate a quantitative enzyme-linked immunosorbent assay (ELISA) for quantifying total IgG (independent of antigenic specificity) in mink serum and milk. The assay allows the study of the dynamics of the exchange of immunoglobulin between mothers and their suckling kits and its significance for protecting against infectious disease to be studied in detail [7].

## Methods

### Sample collection

Forty seven first-year American mink (*Neovison vision*) adult females of mixed genotypes, and their suckling kits (4–12 kits/litter) were haphazardly selected by the farmer from three commercial certified Aleutian mink disease virus (AMDV)-free Danish mink farms (A, B, and C, see Table 1). Farm B and C vaccinated all minks in the summer period (June and July) with a commercial combination mink vaccine, Febrivac de vet (ATC-code#QI20CH, Nordvacc, Greve, Denmark) against mink distemper and mink parvovirus, while farm A did not vaccinate. The minks were housed in separate cages with conventional nest boxes and fed a commercial mink diet with free access to water. The sampling scheme is outlined in Table 1. For studying the linearity under dilution and possible within litter effect of serum IgG concentrations, non-stabilized blood samples from eight adult mink females were taken after euthanasia and all the kits in the litter were euthanized and bled (for one female all kits were taken by Caesarian section after euthanasia and the kits were euthanized and blood sampled immediately). The other 39 adult females and their litters were sampled once a week over a 3 week period by euthanizing and bleeding two of the suckling kits at each blood sampling occasion (blood from the female was taken from the vena cephalica). In connection with blood sampling, females were injected with 0.5 mL of oxytocin (10 IE/mL, #444687, MSD animal health, Copenhagen, Denmark) and maternal milk was obtained by hand-milking. The suckling mink kit blood samples were obtained from kits between 0 and 23 days of age. Blood was allowed to clot and serum was obtained as the supernatant after centrifugation at 4000*G* for 15 min at 4 °C. Serum and milk samples were stored at − 20 °C until analysis.

**Table 1:** Blood samples and milk samples taken during year 2015 and 2016

Year	Farm	Adult females	No. adult female blood samples	No. milk samples	No. kit blood samples	Kit age
2015	A	4	4	4	4–12	0–1 weeks
2015	B	4	4	4	4–12	0–1 weeks
2016	C	20	60^a^	40^a^	120^b^	0–23 days
2016	B	19^c^	57^a^	57^a^	112^b^	0–23 days

^a^Samples taken once a week for 3 weeks

^b^Two kits were sampled from each litter once a week

^c^One mother lost all her kits and was excluded from the study and one litter lost all kits after second week of sampling

### Sandwich ELISA development

#### Purification and use of IgG from pooled mink serum

**Fig. 1:**
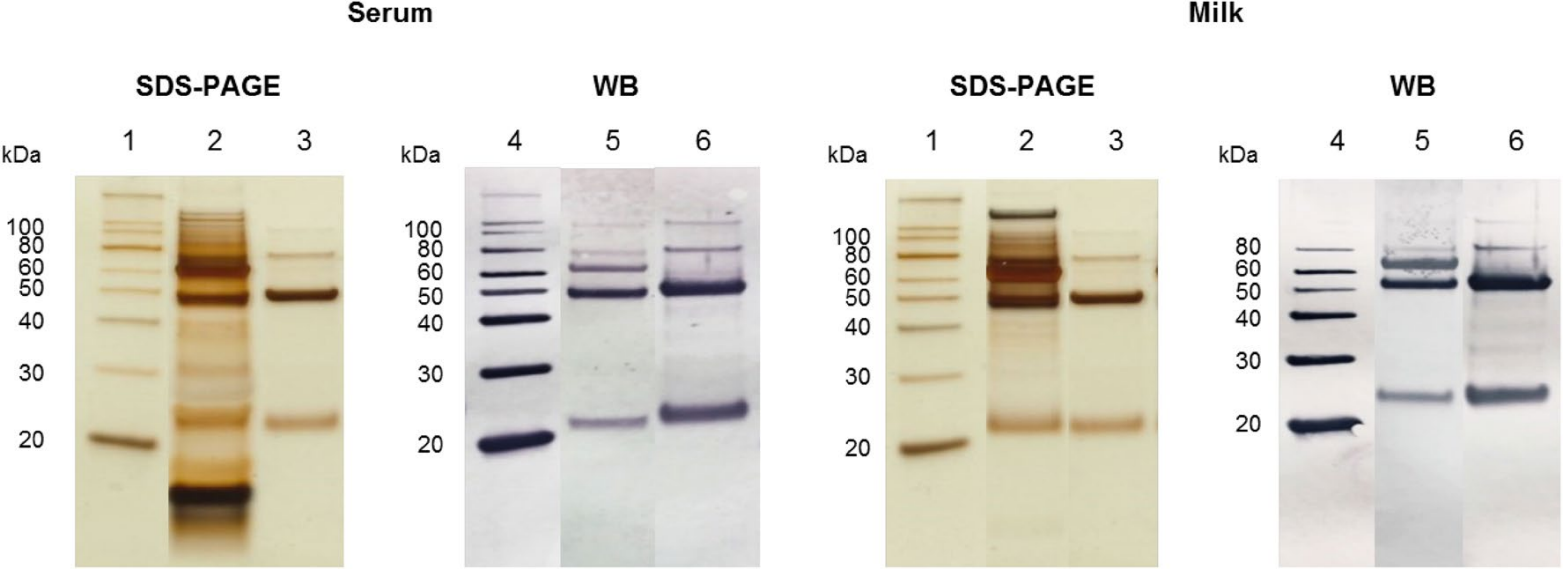
SDS-PAGE and Western blot analysis of a serum sample (left) and milk sample (right), as indicated. The serum sample was diluted 1:1600 and the milk sample was diluted 1:40. In both cases, the purified standard mink IgG preparation was also included. All samples were electrophoresed under reducing conditions on 12% NuPAGE Bis–Tris gel as described in “Methods” section. For Western blotting (WB), the blot was developed with polyclonal goat anti-ferret IgG, followed by alkaline phosphatase-conjugated rabbit anti-goat antibody (see “Methods” section). SDS-PAGE: Lane 1: molecular weight marker; Lane 2: serum/milk sample; Lane 3: purified standard mink IgG. WB: Lane 4: molecular weight marker; Lane 5: serum/milk sample; Lane 6: purified standard mink IgG. The positions of the molecular weight marker proteins (20–100 kDa) are indicated to the left of the gels and blots

Five milliliter of mink serum pooled from eight adult females was mixed with 0.24 g NaCl, 0.155 g glycine and 0.01 g NaOH and passed through a column packed with 5 mL of Protein G Sepharose (GE Healthcare, Bio-Sciences, Uppsala, Sweden). After extensive washing with washing buffer (1.65 M glycine/0.2 M NaOH, 3.3 M NaCl, pH 8.8), elution of IgG was performed with elution buffer (0.1 M glycine/HCl pH 2.8). The absorbance at 280 nm of the eluted IgG fractions was determined on a Nanodrop spectrophotometer (Thermo Scientific, Waltham, MA, USA) and the concentration of IgG in the eluted fractions was calculated assuming a mass extinction coefficient E_280_ of 1.37 at 280 nm for a 1 mg/mL IgG solution. Eluted IgG fractions were pooled and dialyzed against PBS overnight at 4 °C and then analyzed by SDS–polyacrylamide gel electrophoresis (SDS-PAGE) (12% Bis–Tris NuPAGE, Life Technologies, Taastrup, Denmark) followed by silver staining to estimate purity (> 90%, Fig. 1). The IgG concentration of the final mink IgG preparation was estimated by spectrophotometry as described above and the preparation was stored in aliquots at − 20 °C until being used as the standard for the ELISA assay. When applied to the ELISA plate the IgG preparation (standard) was diluted to 0.5 µg/mL with either, 0.05 M Na–acetate buffer, pH 5.5 for serum sample analysis, or 1% w/v casein buffer (1 M NaOH (cat# 106498, Merck)/casein Hammerstein (cat# 440203H, VWR, Radnor, PA, USA)) for milk sample analysis.

#### ELISA protocol for serum and milk samples

96-well Maxisorp polystyrene plates (Nunc, Roskilde, Denmark) were coated overnight at 4 °C with 0.5 µg/mL capture antibody (polyclonal goat anti-ferret IgG (cat# SAB3700792, Sigma-Aldrich, St. Louis, MO, USA) diluted 1:2000 in 0.1 M carbonate buffer (pH 9.6). The antibody is known to cross-react with mink IgG [8]. This and all other steps, except washing steps and blocking, were done with 100 µL solution. After coating, the wells were washed four times with washing buffer, PBS-T (phosphate buffered saline (PBS) containing 0.05% (v/v) Tween 20) filling all wells completely each time (washing procedure). Plates were then blocked for 1 h at room temperature at constant shaking by adding either 200 µL, 1% bovine serum albumin (Sigma-Aldrich) in PBS-T for serum samples or 200 µL, 1% w/v casein buffer [1 M NaOH (cat# 106498, Merck)/casein Hammerstein (cat# 440203H, VWR, Radnor, PA, USA) at 100 mL PBS containing 0.04% (v/v) Tween 20] for milk samples. After another washing procedure, samples were diluted (1:5000-1:6,400,000) and mink IgG standard (see above) was twofold diluted with 0.05 M Na–acetate, pH 5.5 for determination of serum samples, while for measurement of milk samples, milk was diluted (1:30,000) and the IgG standard was twofold diluted with 1% casein buffer. All samples were incubated for 1 h at room temperature with constant shaking. Plates were washed again followed by incubation with horseradish peroxidase (HRP)-conjugated polyclonal goat anti-ferret IgG (cat# SAB3700794, Sigma-Aldrich) diluted 1:800 in blocking buffer for 1 h at room temperature, with constant shaking. After subsequent washing as above, plates were developed with 3,3′,5,5′-tetramethylbenzidine substrate in the presence of hydrogen peroxide (TMB PLUS2, Kem-En-Tec, Taastrup, Denmark). The reaction was stopped after 3–4 min with 0.5 M sulfuric acid. Absorbance was read at 450 nm correcting for background at 650 nm using an automatic plate reader (Thermo Multiskan Ex spectrophotometer, Thermo Scientific, Waltham, MA, USA). All serum and milk samples as well as standard dilutions were run in duplicate determinations.

#### Calculation of sample IgG concentrations

**Fig. 2:**
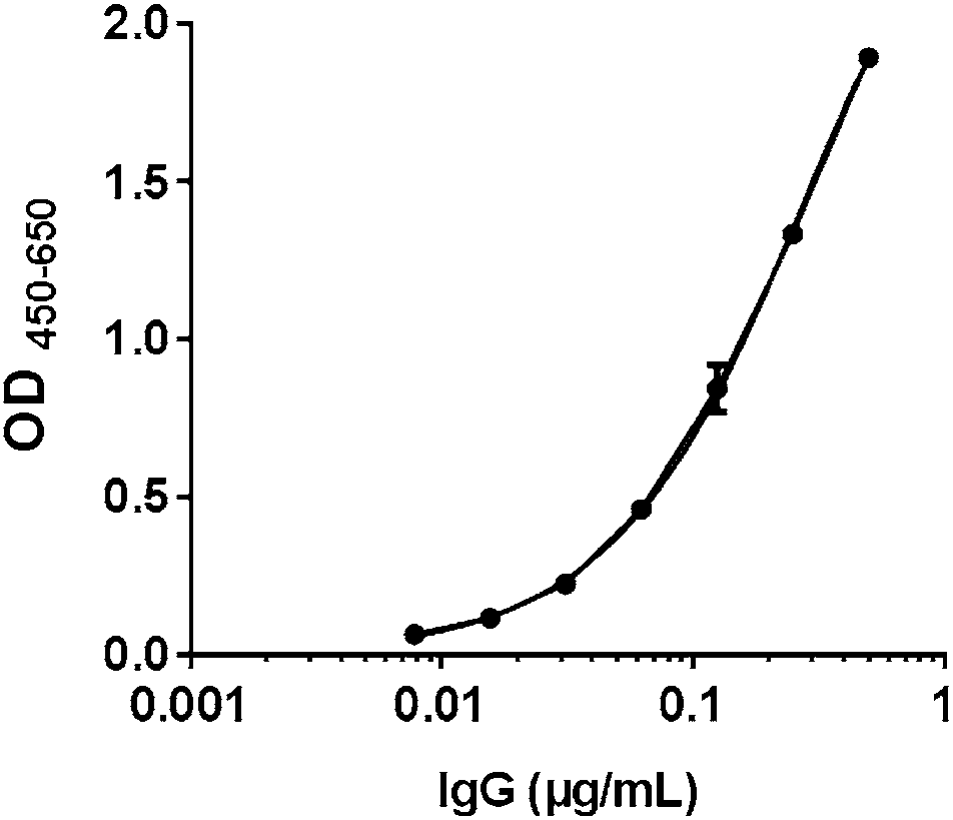
Representative standard curve with seven twofold dilutions of the purified mink IgG standard fitted using a four-parametric logistic curve fit (y = b + (a − b)/(1 + xc)^d and R^2^ = 0.998). The y-axis displays the absorbance at 450 nm after subtraction of the absorbance at 650 nm. Mean ± SD for duplicate determinations of each concentration is indicated on a log-scale

The standard curve (Fig. 2) was constructed from a seven point twofold dilution series of the purified mink IgG standard using a four-parametric logistic curve fit [Ascent software v. 2.6 (Thermo Scientific, Waltham, MA, USA)]. Concentrations of mink serum and milk samples were interpolated and calculated from the within-assay standard curve. To account for background signal all absorbance readings were adjusted by subtraction of the mean absorbance of wells not containing sample (blanks with only buffer).

### Validation

Specificity, detection limit, precision (intra-assay CV and inter-assay CV), linearity under dilution, and limit of quantification for the mink IgG sandwich ELISA were determined for mink serum and milk.

#### Specificity analysis by electrophoresis and Western blot

The purified mink IgG standard, serum, and milk were mixed with 1/4 LDS sample buffer and 1/10 reducing agent (Life Technologies) before being loaded onto a well on an SDS-PAGE Novex NuPAGE 12% Bis–Tris gel (Ref# NP0341BOX, Invitrogen, Carlsbad, CA, USA). NuPAGE MES buffer system was used according to manufacturer’s instructions (Life Technologies) and the gel was run at 200 V constant current. After electrophoresis the samples/bands were visualized using silver staining. In the immunoblot analysis, the purified mink IgG standard, serum, and milk were transferred, after being separated by SDS-PAGE, by electrophoresis from the SDS-PAGE gel onto a nitrocellulose membrane (cat# 5045A330R, Advantec MFS, Dublin, Canada) using a Mini Trans-Blot transfer cell (Bio-Rad, Copenhagen, Denmark) filled with transfer buffer (12.5 mM Tris, 96 mM glycine, 20% ethanol, pH 8.4), for 1 h at 150 mA constant current. Subsequent steps were done at room temperature and with shaking, unless stated otherwise. The membrane was blocked using TBS-T (50 mM Tris, 250 mM NaCl with 0.1% Tween 20) with 2% Tween 20 for 10 min; followed by 3 × 5 min washes in TBS-T. After washing, the membrane was incubated with polyclonal goat anti-ferret IgG (cat# SAB3700792, Sigma-Aldrich) diluted 1:500 in TBS-T, at 4 °C with shaking overnight. The membrane was then washed 3 × 10 min with TBS-T before the last incubation for 1 h with alkaline phosphatase-conjugated rabbit anti-goat antibody (cat# A4062, Sigma-Aldrich) diluted 1:2000 with TBS-T. After 3 × 10 min washes with TBS-T the membrane was developed with 4-nitro-blue-tetra-zolium/5-bromo-4-chloro-3-indolyl-phosphate (NBT/BCIP) tablets (cat# 11697471001, Roche, Hvidovre, Denmark) following the manufacturer’s instructions. Color development was terminated by washing the membrane with several changes of Milli Q water.

#### Detection limit

The lower limit of detection was calculated from the mean absorbance of 12 replicate readings of blank samples (buffer only) to which three standard deviations (SD) were added. In addition, the lower limit of quantification was calculated from the mean absorbance of 12 replicate readings of blank samples plus 10 SD. The corresponding IgG concentration was extrapolated from the standard curve [9].

#### Intra- and inter-assay coefficient of variation

Intra-assay CV was determined by measuring the absorbance of 12 replicates of one mink serum and one mink milk sample in a seven twofold dilution series within the same assay. The inter-assay CV for mink serum was determined by measuring the absorbance for 12 replicates of one mink serum sample in a seven twofold dilution series on five different plates on 2 different days, generating a total of 10 runs. Inter-assay CV for mink milk was determined by measuring the absorbance for 12 replicates of one mink milk sample in a seven twofold dilution series on two different plates on 3 different days, generating a total of six runs. Uncalibrated mean absorbance values were used to calculate the CV (CV% = (SD/mean) * 100) for all the dilutions. A CV value of less than 10% (intra-assay CV) and 15% (inter-assay CV) was regarded as acceptable [10].

#### Linearity of dilution for serum samples

Serum samples from four adult mink females and one mink kit were analyzed in twofold dilution series with a starting dilution of 1:100,000 and 1:30,000, respectively. The four female and one mink kit serum samples were selected to cover a wide range of IgG concentrations.

#### Linearity of dilution for milk samples

Four milk samples were applied in twofold dilution series with a starting dilution of 1:4000 to validate linearity of dilution for mink milk samples.

### Statistical methods

Data was transferred to GraphPad Prism version 7 (GraphPad Software, San Diego, CA, USA, http://www.graphpad.com) for graphic representation and for statistical analysis. Variances within litters and between litter means were compared using the Browne–Forsythe test of equality of variance in Prism. First, the within-litter variance was compared between litters to determine if the variance was different from one litter to the other. Then these variances were compared with the variance of the means of the litters, using the same test, in order to determine if the variance of the mean was different from the within-litter variance. Based on the data in Fig. 6b, looking at means and standard deviations for each of the ages investigated, a sample size of n = 4 was decided as adequate (with a power of 80%) to detect a 1.6-fold difference in serum IgG concentrations of two randomly chosen litters with a statistical significance of 0.05.

## Results

### Specificity

By SDS-PAGE and Western blotting analyses the specificity of the antibody used to detect IgG in both serum and milk was investigated, as illustrated in Fig. 1. It is clearly seen that the antibody reacts with IgG light and heavy chains and no other proteins in either serum or milk, except for a slight non-specific binding to a major protein in the 65 kDa range, most probably serum albumin.

### Intra- and inter-assay variation and detection limit

A representative standard curve for purified mink IgG is depicted in Fig. 2 showing a dynamic range of approximately 2 logs and a sigmoid shape as expected from an ideal standard curve. The operational range of the standard curve was 0.008–0.05 µg/mL. As shown by Table 2 the range of the serum intra-assay CV was 2.15–5.97%, while the range of the inter-assay CV was between 5.17 and 17.78%, with seven twofold dilutions starting at 100,000. In addition, Table 2 also depicts the range of milk intra-assay CV, which was 2.71–5.92%, while the range of the inter-assay CV was 4.20–16.03%, with seven twofold dilutions starting at 30,000. Using the mean optical density of 12 blank replicates containing only buffer plus three times the SD, the lower limit of detection was calculated to 5 ng/mL for serum samples and 1 ng/mL for milk samples. The lower limit of quantification was calculated by using the mean optical density of 12 blank replicates plus 10 times the SD and the results for serum samples was 11 and 3 ng/mL for milk samples.

**Table 2:** Intra- and inter-assay variation for both serum and milk samples

	*n*	Serum		%CV	*n*	Milk		%CV
		OD_450−650 nm_				OD_450−650 nm_		
		Mean	SD			Mean	SD	
Intra-assay variation	12	1.97	0.04	2.15	12	2.15	0.06	2.71
	12	1.42	0.03	2.41	12	1.63	0.05	3.27
	12	0.84	0.02	2.44	12	1.05	0.04	3.51
	12	0.54	0.02	3.71	12	0.66	0.02	3.35
	12	0.37	0.01	3.79	12	0.40	0.01	2.91
	12	0.27	0.02	5.97	12	0.28	0.01	4.15
	12	0.23	0.01	4.48	12	0.22	0.01	5.92
Inter-assay variation	2	2.40	0.35	14.75	3	2.09	0.09	4.20
	2	1.80	0.32	17.78	3	1.58	0.09	5.55
	2	1.01	0.14	14.12	3	1.04	0.09	8.43
	2	0.62	0.07	10.98	3	0.64	0.05	7.94
	2	0.41	0.03	7.68	3	0.36	0.05	12.99
	2	0.29	0.02	6.22	3	0.25	0.03	12.14
	2	0.24	0.01	5.17	3	0.19	0.03	16.03

The intra-assay variation of the serum and milk IgG sandwich ELISA was determined using 12 replicates of seven dilutions of the sample (n = 12). Inter-assay variation was determined by performing 12 replicates of seven dilutions of either one serum sample on five different plates measured on 2 different days (n = 2) or one milk sample on two different plates measured on 3 different days (n = 3). *SD* standard deviation, *CV* coefficient of variation

### Linearity of dilution

**Fig. 3:**
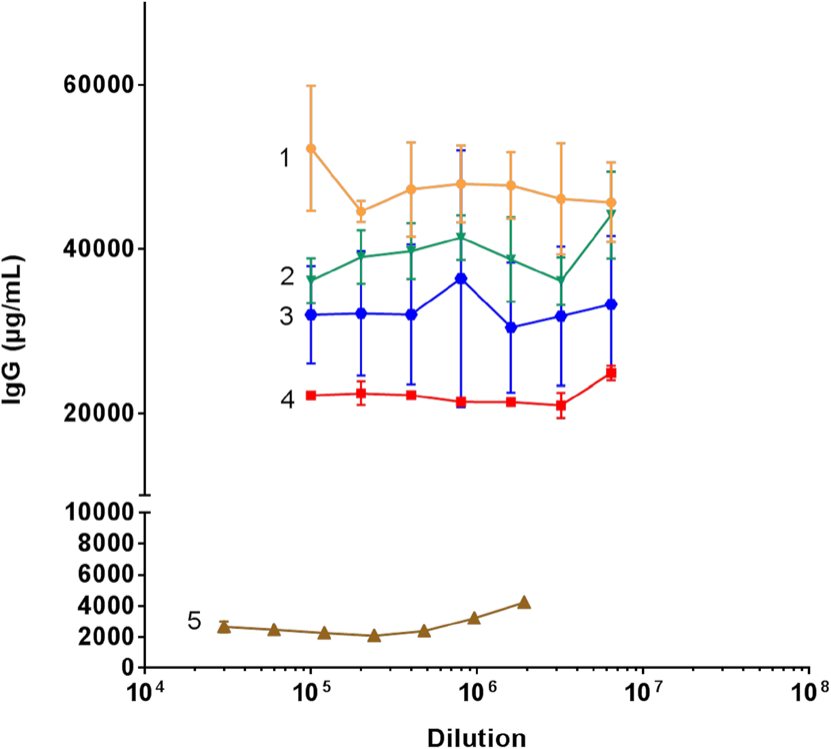
IgG concentrations of four different adult female mink sera (1–4) and one mink kit (5) diluted in sodium acetate buffer as indicated and assayed in duplicates. The results are shown as mean ± SD and the dilutions are shown on a log scale

**Fig. 4:**
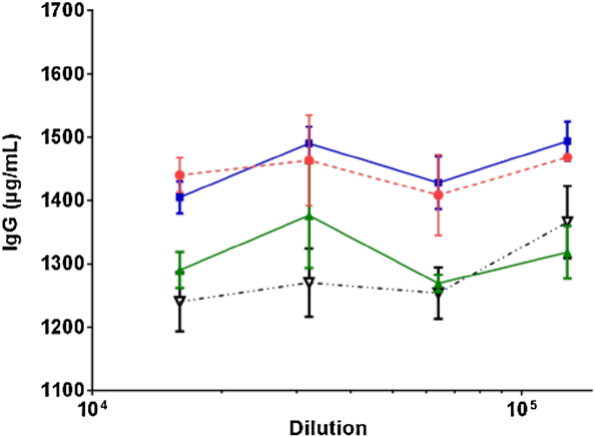
IgG concentrations of four different adult female mink milk samples with dilutions ranging from 16,000 to 128,000. All samples were diluted in 1% casein buffer and run in duplicates. The results are shown as mean ± SD and the dilutions are shown on a log scale

IgG concentrations of five mink sera with dilutions ranging from 30,000 to 6,400,000 were analyzed using the sandwich ELISA. For all sera almost identical concentration values were found at almost all dilutions for each of the sera, with no trend deviating from the value (as indicated by the horizontal lines in Fig. 3), although three non-systematic deviations were noted for serum samples 1, 2, and 3 (Fig. 3). Four milk samples were assayed in twofold dilutions with dilutions starting from 16,000 to 128,000, and as depicted in Fig. 4 the calculated milk IgG concentration was not affected noticeably by dilution. Thus, for both serum and milk the assay was linear over approximately 2 logs.

### Serum IgG concentration of litters

**Fig. 5:**
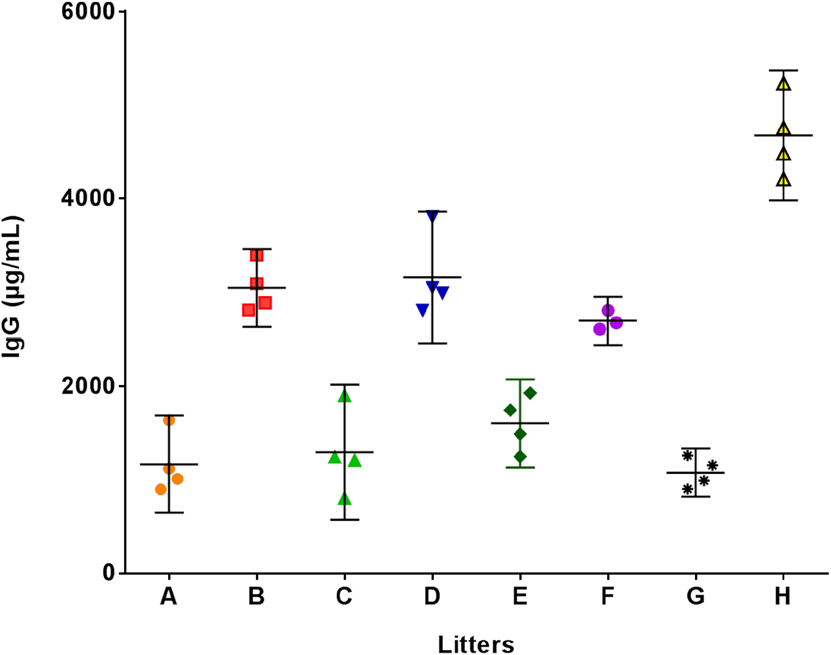
Scatter plot showing mink kit serum IgG concentrations in the litters investigated (n = 3–4 from each litter). All samples were diluted 1:30,000 in sodium acetate buffer and run in duplicates. Brown–Forsythe test of equality of variance was used to observe statistical significance in variances between litters and within the litters (P = 0.0013)

IgG concentration was determined in serum samples from three to four kits taken from eight different litters, by the validated sandwich ELISA. A litter specific effect with respect to serum IgG concentrations was clearly seen, i.e. the within-litter variance in serum IgG concentration was not statistically significantly different between litters, while these variances were statistically significantly different from the variance between mean serum IgG concentrations of the litters (P = 0.0013, Brown–Forsythe test of equality of variance). The variance between litter means was higher than the variance within litters (Fig. 5).

### Serum IgG concentrations of mothers and their kits

**Fig. 6:**
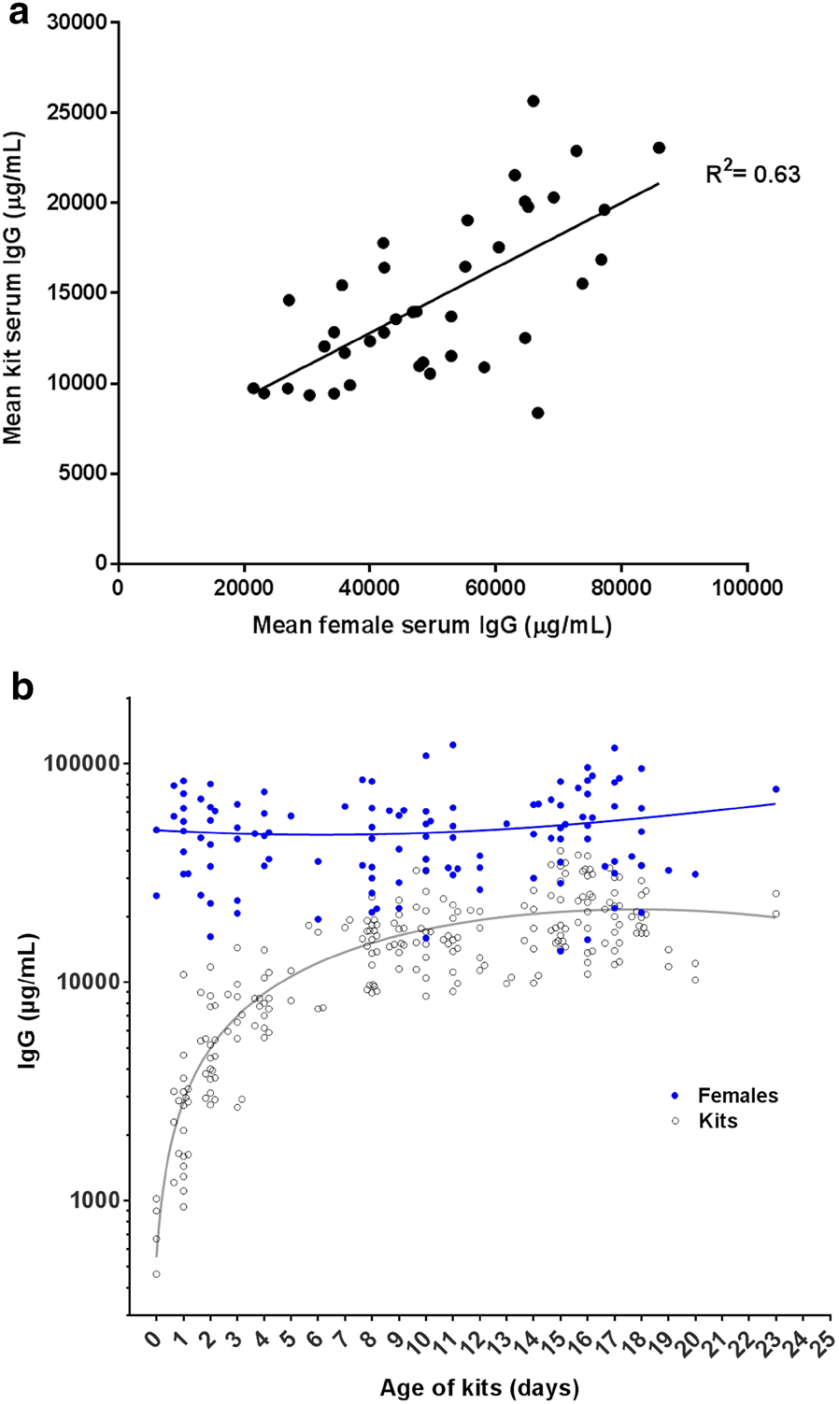
**a** Scatter plot and regression curve of mean kit IgG versus mean maternal IgG concentrations. Mean kit concentrations were derived from the serum IgG concentrations of four to six kits from a specific litter analyzed over a 3 week period (2 kits each week) and corresponding mean maternal/female concentrations were obtained from three samples taken over the same 3 week period. Positive correlation (R^2^ = 0.63, P < 0.0001) of mean kit serum IgG concentration and mean maternal/female serum IgG concentration is shown on the scatter plot. **b** Scatter plot diagram showing maternal/female serum IgG concentrations (blue dots) and mink kit IgG serum concentration (clear dots) as a function of age. The results are expressed as mean ± SEM, with a non-linear curve fit (grey curve for kits and blue curve for maternal/females), and the serum IgG concentrations are show on a log scale. All serums samples were diluted in sodium acetate buffer and assayed in duplicates

A scatter plot with the mean IgG concentrations of the three serum samples from each mother and the mean of the 4–6 corresponding offspring samples is shown in Fig. 6a. Spearman’s correlation coefficient was 0.63 (P < 0.0001) indicating a positive correlation between maternal and kit mean serum IgG concentrations. The majority of maternal IgG serum concentrations were higher than those observed in the kits (Fig. 6b). The kits serum IgG concentration range, over 8 days of age, was 8400–26,000 µg/mL and the maternal serum IgG concentration range was between 13,900 and 109,000 µg/mL. Over time, starting at birth and ending around 8 days after birth an exponential increase in serum IgG concentration was seen in the kits (grey curve in Fig. 6b), while maternal serum IgG stayed almost constant in the same period (blue curve, Fig. 6b).

## Discussion

When studying the possible significance of IgG, with respect to protecting mink kits against various pathogens and diseases, it is important to have a reliable analytical method for analyzing the concentration of IgG in milk and blood. In the present study, the development and validation of a sandwich ELISA for quantifying IgG in mink serum and milk was described. We analyzed specificity, intra-and inter-assay variation, limit of detection, limit of quantification, and linearity of dilution. The specificity of the antibody used in the ELISA was elucidated by investigating its reactivity with full mink serum as well as mink milk by Western blotting. Except from a minor non-specific reaction with the albumin band in both serum and milk samples the antibody reacted exclusively with mink IgG light and heavy chains. The nonspecific binding to albumin may be an artefact related to the blotting procedure and did not present a problem during ELISA analysis as the ELISA background was low. The validation demonstrated that the assay is reproducible (low intra- and inter-assay variation), and sensitive with a lower limit of detection allowing minimal sample volumes (5 µL) to be used. Mean intra-assay CV for serum samples was 3.6 and 3.7% for milk samples and the mean inter-assay CV for serum samples was 11.0 and 9.6% for milk samples, which can be considered acceptable [10]. The lower limit of detection was 5 ng/mL and the limit of quantification was 11 ng/mL for serum samples, which is more than sufficient for measurement of mink serum concentrations of IgG as the lowest serum concentrations of IgG we analyzed in mink kits was 0.4 mg/mL and others have previously reported serum concentrations in the range of 0.1–0.73 mg/mL [1]. Furthermore, milk samples showed a lower limit of detection of 1 ng/mL and a limit of quantification of 3 ng/mL, which is also more than sufficient for milk samples as the lowest milk IgG concentration we analyzed from milk was 1600 µg/mL and others have reported milk IgG concentrations in the range of 1–6.3 mg/mL [1]. It is important for the precision of an ELISA to show good linearity of dilution, i.e. the calculated concentration of IgG in a given sample (within the linear range of the assay) should not be affected by the dilution at which the sample is tested [11]. As shown in Figs. 3 and 4, comparable values were obtained irrespectively of dilution for both serum samples (disregarding a few (3) non-systematically deviating samples) and milk samples indicating lack of interference by non-relevant matrix components. Additionally, an ideal ELISA assay must be robust and should not be affected by small changes in the procedure [11]. The assay was robust as measurements were unaffected by change of operator and as it performed well with analytes stored at − 20 °C for a longer period of time. Also, repeated freezing and thawing did not affect the readout. One limitation of the assay is that no calibrated standard mink IgG was available, thus precluding a definitive calibration of the assay. Here, a preliminary calibration of the purified mink IgG was achieved by spectrophotometry at 280 nm using the generally accepted mass extinction coefficient of 1.37 per 1 mg IgG/mL. Furthermore, in comparison with other studies regarding the concentration of IgG in adult female and kit serum and female milk, the results correspond well with our data [1, 12]. To our knowledge, no ELISA has previously been thoroughly validated for mink IgG quantification in serum and milk samples although a number of older studies report on IgG concentrations in mink blood and milk using non-validated methods. Porter et al. [2] used immunoelectrophoresis and a polyclonal rabbit anti-mink serum antibody to study differences in concentrations of gamma-globulins in maternal vs. offspring serum and in colostrum vs. adult serum [2] while Coe and Race [1] used single radial immunodiffusion and an in-house polyclonal antiserum against mink IgG to quantify mink IgG in serum samples. These two studies show conflicting results with regard to IgG concentrations in newborn kits; Porter et al. [2] indicate that there is no uptake of IgG from the mother's placenta while Coe and Race [1] show that there is indeed uptake of IgG, allowing the newborn kit to have circulating IgG. The present study indicates trans-placental transfer of IgG, as serum IgG was detected in litter C, which was obtained by post mortem Caesarian section (Fig. 5). Other studies have also utilized ELISA for quantification of IgG in mink blood samples [13–15] and mink milk [12]. Previously described ELISAs [13, 14] employed an in-house absorbed rabbit anti-mink IgG serum, which is not commercially available. The assay was either not calibrated [13] or was using an undisclosed method for calibrating their mink IgG standard [14]. An indirect ELISA for quantification of hapten-carrier specific IgG from hapten-carrier conjugate immunized adult mink was also reported [15], however no attempt was made to quantify the naturally occurring total IgG population and no validation was described. Another study investigated the transfer of specific IgG from mother to offspring in the fetal stage by using mothers vaccinated with mink enteritis virus (MEV) and then analyzing the milk-derived MEV specific IgG in the kits’ serum, however the analytical method used in this study was not disclosed [12]. Thus, the litter specific serum MEV-specific IgG concentrations could in principle have been due to different efficiency of maternal vaccination from one mink female to the other. In the present study we clearly demonstrate that individual litters have a well-defined litter specific level of total circulating IgG (Fig. 5), which to some extent is associated with the concentration of total IgG in the maternal circulation (Fig. 6a). Differences in serum IgG concentration between litters (high vs. low IgG concentration, Fig. 5) could have consequences for the immune competence and ability to handle pathogens. Litters with low IgG concentrations might be more susceptible to disease, which could explain the observation that only some litters on a farm is affected by outbreaks of “pre-weaning diarrhea” [16]. Serum IgG concentrations in mink kits reached a plateau 8 days after parturition (Fig. 6b), while maternal IgG concentrations remained fairly constant. Looking back to the studies done by Porter et al. [2] and Core and Race [1], both studies also show that kits reach serum IgG concentrations that are similar to the adult female’s serum IgG concentrations 8 days after parturition [1, 2]. Our data thus corroborate the previous data, however with a much higher number of animals included. Furthermore, we analyzed milk samples collected from 19 adult females milked one time/week over a 3-week-period. The milk IgG concentrations did not correlate in any way with serum concentration in the same individual animal (data not shown). Results reported by Uttenthal et al. [12] indicate that the IgG concentrations in both milk and serum from adult females, sampled over a 4-week-period, did not change much over time, which is what we have also seen in our samples (Fig. 6b). The study by Fink et al. [17] shows that mink colostrum and milk have the same chemical composition indicating that the milk does not change much after the mink kits are born. To sum up, the IgG concentrations found in our collected mink blood and milk samples corroborates earlier studies and adds new data, which further validates our ELISA and establishes it as a reliable analytical tool for analyzing IgG concentrations in serum and milk of mink.

## Conclusions

The validated sandwich ELISA presented here is a sensitive and reproducible calibrated assay suitable for analyzing total IgG concentrations in mink blood and milk. Serum IgG concentrations in mink kits are shown to be litter specific. In addition, a positive correlation between mean maternal serum IgG concentrations and mean kit serum IgG concentrations was established and it is confirmed that mink kit serum IgG concentrations reaches a plateau within 8 days after parturition. Our results corroborate and extent previous investigations, stressing the usability of the ELISA, paving the way for more investigations into the importance of maternal IgG for the welfare and health of the offspring.
